# Factors associated with nursing diagnoses in chronic kidney patients: a cross-sectional study

**DOI:** 10.15649/cuidarte.2160

**Published:** 2021-08-20

**Authors:** André Emanuel Dantas-Mercês, Christielle Lidianne Alencar-Marinho, Flávia Emília Cavalcante Valença-Fernandes, Evanilda Souza de Santana-Carvalho, Wilson Cañon-Montañez, Rudval Souza-da-Silva

**Affiliations:** 1 RN, University of State of Bahia (UNEB/7), Senhor do Bonfim, BA, Brazil. E-mail: med.merces@gmail.com University of State of Bahia Brazil med.merces@gmail.com; 2 RN, MSc, is an Auxiliar Professor at Nursing Collegiate, University of State of Bahia (UNEB/7), Senhor do Bonfim, BA, Brazil. E-mail: christiellealencar@yahoo.com.br University of State of Bahia Brazil christiellealencar@yahoo.com.br; 3 RN, PhD, is an Adjunct Professor at Nursing Collegiate, University of Pernambuco - Petrolina Campus, Petrolina, PE, Brazil. E-mail: flavia.fernandes@upe.br University of Pernambuco Brazil flavia.fernandes@upe.br; 4 RN, PhD, is a Full Professor State University of Feira de Santana. Feria de Santana, BA, Brazil and Permanent Professor of the Postgraduate Program in Nursing and Health at the Federal University of Bahia. E-mail: evasscarvalho@uefs.br University of Bahia Brazil evasscarvalho@uefs.br; 5 RN, PhD, is an Associate Professor at Faculty of Nursing, Universidad de Antioquia, Medellín, Colombia. E-mail: wilson.canon@udea.edu.co Universidad de Antioquia Universidad de Antioquia Colombia wilson.canon@udea.edu.co; 6 RN, PhD, is an Adjunct Professor at Nursing Collegiate, University of State of Bahia (UNEB/7), Senhor do Bonfim, BA, Brazil and Permanent Professor of the Postgraduate Program in Nursing and Health at the Federal University of Bahia. E-mail: rudsouza@uneb.br Federal University of Bahia Brazil rudsouza@uneb.br

**Keywords:** Nursing Process, Nursing Diagnosis, Renal Insufficiency, Chronic, Renal Dialysis., Proceso de Enfermería, Diagnóstico de Enfermería, Insuficiencia Renal Crónica, Diálisis Renal., Processo de Enfermagem, Diagnóstico de Enfermagem, Insuficiência Renal Crônica, Diálise Renal

## Abstract

**Introduction:**

To the care plan, the nurses must use the nursing process and adopt their perspectives, cognitive and documentary, considering the human responses of each chronic kidney disease patient, todefinethenursingdiagnoses, results, andinterventions.This study is aimed to analyze the nursing diagnoses of NANDA-I in chronic renal patients and its association with clinical and sociodemographic variables.

**Materials and Methods:**

Analytical cross-sectional study, performed with chronic renal patients undergoing hemodialysis. The study population consisted of 177 medical records of chronic kidney disease patients. They were selected in the pre-established period of six months: July to December 2018. It was used the Google Forms® platform to import the data directly to Microsoft Excel® Program by generating an electronic spreadsheet that allowed organizing the data, this was then transcribed to the Stata 14.0 software to perform thestatisticalanalyses.

**Results:**

Theriskforbleedingdiagnosisshowed a significant difference with the variable access route (p = 0.02); risk for falls was associated with the variables age, occupation, education (p <0.01) and excessive fluid volume with the variable duration of treatment (p = 0.01).

**Discussion:**

In the case of nursing diagnoses, these must be planned and documented based on a standardized nursing language, with NANDA-I.

**Conclusion:**

The findings of this study showed the main nursing diagnoses and its association with clinical and sociodemographic variables in chronic renal patients in a Brazilian context. Future research may lead to implement care plans for the most prevalent nursing diagnoses in this type of population.

## Resumen

**Introducción:**

Al plan de cuidados, los enfermeros deben utilizar el proceso de enfermería y adoptar sus perspectivas, cognitivas y documentales, considerando las respuestas humanas de cada paciente con enfermedad renal crónica, para definir los diagnósticos, resultados e intervenciones de enfermería. Este estudio tiene como objetivo analizar los diagnósticos de enfermería de la NANDA-I en pacientes renales crónicos y su asociación con variables clínicas y sociodemográficas.

**Materiales y Métodos:**

Estudio analítico de corte transversal, realizado con pacientes renales crónicos en hemodiálisis. La población de estudio consistió en 177 prontuarios de pacientes con enfermedad renal crónica. Fueron seleccionados en el período preestablecido de seis meses: julio a diciembre de 2018. Se utilizó la plataforma Google Forms® para importar los datos directamente al programa Microsoft Excel® mediante la generación de una hoja electrónica que permitió organizar los datos, esta fue luego transcrito al software Stata 14.0 para realizar los análisis estadísticos.

**Resultados:**

El diagnóstico riesgo de sangrado mostró una diferencia significativa con la variable vía de acceso (p = 0.02); riesgo de caídas fue asociado con las variables edad, ocupación, educación (p <0.01) y volumen excesivo de líquidos con la variable duración del tratamiento (p = 0.01).

**Discusión:**

En el caso de los diagnósticos de enfermería, estos deben ser planificados y documentados con base en un lenguaje estandarizado de enfermería, con NANDA-I.

**Conclusión:**

Los hallazgos de este estudio mostraron los principales diagnósticos de enfermería y su asociación con variables clínicas y sociodemográficas en pacientes renales crónicos en un contexto brasilero. Las investigaciones futuras pueden llevar a implementar planes de cuidado para los diagnósticos de enfermería más prevalentes en este tipo de población.

## Resumo

**Introdução:**

No plano de cuidados, o enfermeiro deve utilizar o processo de enfermagem e adotar suas perspectivas, cognitiva e documental, considerando as respostas humanas de cada paciente com doença renal crônica, para definir os diagnósticos, resultados e intervenções de enfermagem. Este estudo teve como objetivo analisar os diagnósticos de enfermagem da NANDA-I em pacientes renais crônicos e sua associação com variáveis clínicas e sociodemográficas.

**Materiais e Métodos:**

Estudo transversal analítico, realizado com pacientes renais crônicos em hemodiálise. A população do estudo consistiu em 177 prontuários de pacientes com Doença Renal Crônica, os quais foram selecionados num período pré-estabelecido de seis meses: julho a dezembro de 2018. Foi utilizada a plataforma Google Forms® para importar os dados diretamente para o programa Microsoft Excel® gerando uma planilha eletrônica que permitiu a organização dos dados, em seguida transcrito para o software Stata 14.0 para realizar as análises estatísticas.

**Resultados:**

O diagnóstico risco de sangramento apresentou diferença significativa com a variável via de acesso (p = 0,02); risco de quedas foi associado às variáveis idade, ocupação, escolaridade (p <0,01) e volume excessivo de líquidos com a variável tempo de tratamento (p = 0,01).

**Discussão:**

No caso de diagnósticos de enfermagem, estes devem ser planejados e documentados com base em uma linguagem de enfermagem padronizada, com a NANDA-I.

**Conclusão:**

Os achados deste estudo evidenciaram os principais diagnósticos de enfermagem e sua associação com variáveis clínicas e sociodemográficas em pacientes renais crônicos no contexto brasileiro. Pesquisas futuras podem levar à implantação de planos de cuidados para os diagnósticos de enfermagem mais prevalentes nesse tipo de população.

## Introduction

Chronic Kidney Disease (CKD) is a common clinical condition that has been increasing daily across the world. Its causal factors are as follows: the aging population, and the high growth of risk conditions, such as obesity. It is a clinical situation that has implications in both the personal lives of each individual, and in public health systems, burdening public coffers with the costs of treatment(1).

With regard to developing countries, the situation becomes more critical in consideration of worldwide socially disadvantaged populations, where there is a greater consequence of kidney disease, and thus its ensuing burdens; as well as the diversity of conditions in the provision of kidney care(2)-(3).

According to the World Health Organization, one of the goals of sustainable development focus on achieving universal health coverage worldwide by 2030, this includes elements related to kidney treatment with the provision of care coverage in all countries; in a quest to reduce of the burden and implications of CKD as an important step towards equity in kidney health(4).

In the care of chronic renal patients, the nurse and their team have an important role, with emphasis being as an educating agent, promoting the exchange of information, together with the implementation of a care plan focused on three interrelated themes, namely: strategies involving family, patient self-care, and integration into society(5).

In order to plan care, nurses must use the Nursing Process and adopt their perspectives, both cognitive and documentary; and consider the human responses of each CKD patient, to define the elements of practice. In the case of nursing diagnoses, these must be inferred and documented based on a Standardized Nursing Language, with NANDA International Taxonomy II used for the study as a classification system for nursing diagnosis(6).

In the scenario of CKD patients, treatment modalities are observed, and hemodialysis is the most common. Knowing the sociodemographic and clinical characteristics of those CKD patients undergoing hemodialysis, and making associations with Nursing Diagnoses that are documented in the patients’ records, creates an understanding of the main human responses affected; also making it possible to identify possible gaps in the Nursing Process, Thus nurses will be made aware of better care planning, and be able to respect the individuality of each patient, as they are supporting better evidence-based practice. The aim of this study was to analyze the nursing diagnoses of NANDA-I in chronic renal patients and its association with clinical and sociodemographic variables.

## Materials and Methods

Cross-sectional analytical study(7), which followed the recommendations of the STROBE statement(8).

The study was carried out from April to July 2019 at an institution specializing in the treatment of CKD patients, it is located in a macro-region of Northeast Brazil. The service is of a private administrative nature, but is part of the Unified Health System, which is public and free of charge. The service offers outpatient medical care, a nursing service, and provides renal replacement therapy (hemodialysis and peritoneal dialysis) care. This is carried out weekly, with approximately 214 patients on hemodialysis, and 10 on peritoneal dialysis, working over three shifts for six days a week.

The nurses assessed the patients at each session and documented their diagnoses and interventions in the patient’s chart every month. However, the nurse recorded clinical changes and variations at each hemodialysis session. For the diagnoses, the nurses used NANDA-I Taxonomy II (2018-2020)(6), and for interventions used a set of nursing actions / interventions divided into two stages of therapy, which were pre- and post-dialysis.

The study sample consisted of 177 medical records of CKD patients. All medical records were selected in the pre-established period of six months: July to December 2018. Whose patient profile according to medical records included in this study were those on haemodialysis treatment for more than six months of treatment, and those who had completed registration of the stages of the nursing process with respective nursing diagnoses and interventions. Patients under the age of 18 or with incomplete information were excluded.

Following the already mentioned criteria, 10 patients in outpatient follow-up were excluded, whose nursing team records did not include the elements of the practice. That way, the study population was of 187 medical records considering the period under analysis.

Data was collected by two trained researchers, under the supervision of an advisor. For data collection, a structured form anchored on the Google Forms® platform was developed by the authors. This contained characterization of the patients as documented in the paper chart, followed by the extraction of nursing diagnoses as defined by the Nursing Care Systematization Commission, presented as a specific instrument to document the Nursing Process, and included in the form.

The Google Forms® platform made it possible to import the data directly to Microsoft Excel® program by generating an electronic spreadsheet that allowed organizing the data, this was then transcribed to the Stata 14.0 software to perform statistical analyses.

To perform the analysis, the normality of interest variables was initially tested using Shapiro- Wilk, to see associations between independent and dependent variables, Pearson’s chi-square tests and Fisher’s exact tests were used for categorical variables. P values <0.05 were considered statistically significant.

The study was approved by the Ethics and Research Committee of the responsible institution, according to opinion nº 3.194.353, this respects national and international standards for research involving humans.

The variables characterizing the sociodemographic profile were age, sex, marital status, religion, occupation, education, and income. For the clinical profile, health history, length of treatment, access to hemodialysis and nursing diagnoses were investigated.

The nursing diagnoses used in the present study, with the respective numerical codes, are described below, separated into risk diagnoses and diagnoses focusing on the problem(6).

Risk Diagnostics: Risk for unstable blood glucose level (0179); Risk for infection (00004); Risk for falls (00155); Risk for allergic reaction (00217); Risk for bleeding (00206). Diagnostics problem- focused: Impaired verbal communication (00051); Feeding self-care deficit (00102); Bathing self-care deficit (00108); Chronic pain (00133); Impaired skin integrity (00046); Imbalanced nutrition: less than body requirements (00002); Excess fluid volume (00026).

## Results

177 patients were investigated by analyzing their medical records, the majority of whom were male (58.8%), with an average age of 54 years, married (60.5%), retired (69.5%), with an income of up to one minimum wage (61.6%). Most were Catholic (74.6%) and had up to nine years of study (85.9%). Regarding the duration of treatment, most (57.1%) had more than 3 years on hemodialysis and used arteriovenous fistula to perform the therapy (84.7%). About the comorbidities, the most (49.7%) did not have them (Table 1).

**Table 1:** Sociodemographic characterization of patients on hemodialysis according to data from medical records. Senhor do Bonfim, BA, Brazil, 2019.

Variable	n = 177	(%)
Age range		
20-40 years	39	22.0
41-60 years	74	41.8
61-80 years	56	31.6
81-99 years	8	4.5
Sex		
Male	104	58.8
Female	73	41.2
Marital status		
With companion	107	60.5
No companion	70	39.5
Religion		
Catholics	132	74.6
Evangelicals	37	20.9
Occupation		
Retired	54	30.5
Others	123	69.5
Education		
Up to 9 years of studies	152	85.9
More than 9 years of studies	25	14.1
Income (per capita salary) *		
0 - 1	109	61.6
2 or more	29	16.4
Health history		
Denies comorbidities	88	49.7
SHA or DM	51	28.8
SHA and DM	38	21.5
Treatment time		
Less than 3 years	76	42.9
Over 3 years	101	57.1
Access way		
Arteriovenous fistula	150	84.7
Others	27	15.3

* Minimum Wage in Brazil in 08/26/2020 $ 189.65 (Dollar Quotation R$ 5.51). SHA: Systemic Arterial Hypertension; DM: Diabetes Mellitus.

Source: research date.

Twelve nursing diagnoses were identified, according to the NANDA-I taxonomy, and among these, the risk of bleeding, risk of falls and excessive fluid volume were the most frequent, presenting in 67.2%, 56.5% and 54.8% of patients according to documentation in medical records, respectively. The average number of diagnoses per patient was 2.61 (Table 2).

**Table 2:** Frequency of nursing diagnoses for patients on hemodialysis according to medical records. Senhor do Bonfim, BA, Brazil, 2019.

Nursing Diagnoses	n = 177	(%)
Risk for bleeding	119	67.2
Risk for falls	100	56.5
Excess fluid volumen	97	54.8
Risk for unstable blood glucose level	52	29.4
Risk for infection	48	27.1
Risk for allergic reaction	21	11.9
Bathing self-care deficit	5	2.8
Chronic pain	4	2.3
Impaired verbal communication	3	1.7
Impaired skin integrity	2	1.1
Feeding self-care deficit	1	0.6
Imbalanced nutrition: less than body requirements	1	0.6

Source: research date.

Table 3 shows the results of the association between the three most frequent diagnoses with clinical and sociodemographic variables. The risk for bleeding diagnosis shows significant differences only when associated with the type of venous access to perform the therapy, so that patients using the arteriovenous fistula had this diagnosis compared to those who used other types of access.

The diagnosis of risk for falls had a significant difference with variables age range, occupation, education, and access method. Patients in the age categories 61-80 and 81-99 years, retired and with up to nine years of study, had the diagnosis of risk for falls compared with those younger people, not retired, and with more than nine years of study.

The diagnosis of excess fluid volume only showed a significant difference with the duration of treatment, so that individuals with more than three years of treatment presented this diagnosis.

**Table 3:** Bivariate analysis of sociodemographic and clinical variables and the three most frequent nursing diagnoses in hemodialysis patients according to medical record data. Senhor do Bonfim, BA, Brazil, 2019.

Variables	Risk for bleeding				Risk for falls				Excess fluid volume			
	YES	NO			p*	YES		NO		p*	YES		NO		p*
	n	%	n	%		n	%	n	%		n	%	n	%	
Age range					0.90					<0.01					0.50**
20-40 years	26	21.8	13	22.4		4	4.0	35	45.5		19	19.6	20	25.0	
41-60 years	48	40.3	26	44.8		34	34.0	40	51.9		43	44.3	31	38.8	
61-80 years	39	32.8	17	29.3		54	54.0	2	2.6		29	29.9	27	33.8	
81-99 years	6	5.0	2	3.4		8	8.0	0	0.0		6	6.2	2	2.5	
Sex					0.53					0.70					0.75
Male	68	57.1	36	62.1		60	60.0	44	57.1		58	59.8	46	57.5	
Female	51	42.9	22	18.5		40	40.0	33	42.9		39	40.2	34	42.5	
Marital Status					0.18					0.42					0.29
With companion	43	36.1	27	46.6		37	37.0	33	42.9		35	36.1	35	43.8	
No companion	76	63.9	31	53.4		63	63.0	44	57.1		62	63.9	45	56.3	
Religion					0.49					0.29					0.61
Catholics	90	79.6	42	75.0		77	81.1	55	74.3		74	79.6	58	76.3	
Evangelicals	23	20.4	14	25.0		18	18.9	19	25.7		19	20.4	18	23.7	
Occupation					0.34					<0.01					0.89
Retired	39	32.8	15	25.9		46	46.0	8	10.4		30	30.9	24	30.0	
Others	80	67.2	43	74.1		54	54.0	69	89.6		67	69.1	56	70.0	
Education					0.19					<0.01					0.24
Up to 9 years of studies	105	88.2	47	81.0		92	92.0	60	77.9		86	88.7	66	82.5	
More than 9 years of studies	14	11.8	11	19.0		8	8.0	17	22.1		11	11.3	14	17.5	
Income (per capita salary) *					0.23					0.66					0.93
0 - 1	22	23.9	7	15.2		16	19.8	13	22.8		16	20.8	13	21.3	
2 or more	70	76.1	39	84.8		65	80.2	44	77.2		61	79.2	48	78.7	
Health history					0.40					0.27					0.27**
Denies comorbidities	55	46.2	33	56.9		53	53.0	35	45.5		47	48.5	41	51.3	
SHA or DM	37	31.1	14	24.1		24	24.0	27	35.1		25	25.8	26	32.5	
SHA and DM	27	22.7	11	19.0		23	23.0	15	19.5		25	25.8	13	16.3	
Treatment time					0.49					0.34					0.01
Less than 3 years	70	58.8	31	53.4		54	54.0	47	61.0		47	48.5	54	67.5	
Over 3 years	49	41.2	27	46,6		46	46.0	30	39.0		50	51.5	26	32.5	
Access way					0.02										
Arteriovenous fistula	106	89.1	44	75.9		80	80.0	70	90.9	0.05	82	84.5	68	85.0	0.93
Others	13	10.9	14	24.1		20	20.0	7	9.1		15	15.5	12	15.0	

Pearson's chi-square; ** Fisher' exact test.

Source: research date.

## Discussion

This study identified the main factors or variables (access route, age group, occupation, education, and length of treatment) associated with the most prevalent NANDA-I nursing diagnoses in CKD patients.

Based on the analysis of the results, it was possible to outline the sociodemographic and clinical profile of renal patients undergoing hemodialysis. With regard to sex, marital status and age range, the results of this study are corroborated by the national sociodemographic pattern described in the Brazilian Dialysis Census(9).

In addition, the studies(10)-(11) demonstrate is a worldwide trend the predominance of male chronic kidney patients, which may be related to the fact that men use for health services for longer periods of time, especially when the disease is silent and in the initial phase.

Low levels of income and education were found in the studies(10),(12)-(13) corroborated by other national and international studies. It is inferred that individuals with unfavorable socioeconomic conditions, such as lower education and income, are more exposed to risk factors for chronic diseases, in addition to a lesser understanding of self-care measures.

Considering the history of comorbidities, hypertension was reported the most, which characterizes a common profile in developing countries, in which access to health services is limited. In countries such as the USA, Mexico and Portugal, diabetes predominates as the most prevalent comorbidity(14).

As for the nursing diagnoses, the average number of these was 2.6 per patient. This is lower than the average for a study carried out in Northeast Brazil, which had an average of 4.6 diagnoses(15).

Of the identified diagnoses, three occurred frequently, these were: risk for bleeding, risk for falls and excess fluid volumes. Such diagnoses signify priority nursing attention with regard to prevention and protection of patients, as well as being diagnoses focused on daily care to patients during hemodialysis sessions(16).

The nurse has a strategic role in establishing nursing diagnoses and to infer from these the correct care planning procedure, it is worth highlighting the importance of individualizing these plans, so as to provide the CKD patient with directed care to their affected human responses(17).

In recent years, more individualized care for renal patients on dialysis has been discussed, seeking to include realistic goals centered on the patient and a shared decision made between the health team, patient, and family. For this assistance to be effective, it must be adapted to the patient’s cognitive function, and their health awareness, in addition to their socioeconomic factors and knowledge and experiences regarding treatment(11).

The development of assistance directed by the Nursing Process is a possibility for individualizing care, it is based on an understanding of the individual’s personal needs, in addition to enabling nurses to use standardized nursing language as a basis for documenting responses and the nursing goals and interventions plans(18).

However, the presence of comorbidities favours an increased risk for these patients, which requires differentiated care. There is a lower level of education associated with the higher number of nursing diagnoses, which may be related to miscomprehension of the guidelines, resulting in difficulty to adhere to therapy. Health education is a therapeutic intervention of extreme importance in that it reduces complications due to comorbidities and the number of hospitalizations, as well as enabling better control of clinical indicators(19).

No reports were identified in the literature associating nursing diagnoses with treatment time. However, it is a fact that when people start dialysis, they show physical signs and symptoms due to uremia, as well as the emotional and social changes inherent in the treatment. Over time, there is a decrease in complications and a better adaptation to the process, contributing to a better health status(20).

The most frequent diagnosis, risk for bleeding, was associated only with the variable type of venous access and is one of the most prevalent nursing diagnoses in studies(21). Although almost studies indicate this diagnosis in 100% of patients, it is important to highlight that this practice can bring problems whereas the cognitive perspective of the Nursing Process, making individualized care planning difficult, whom the survey of a nursing diagnosis requires the presence of clinical evidence to support the diagnostic accuracy.

Patients using Arteriovenous Fistula (AVF) had a higher frequency of the risk for bleeding. This fact is justified by the numerous venipunctures associated with anticoagulation with heparin in the dialysis system, in addition to changes resulting from the pathophysiology of kidney disease itself(21)-(22).

The risk for falls was more presented in elderly patients, those retired, and with a lower level of education. A study with chronic renal patients carried out in a city in the state of São Paulo, Brazil, showed that 93.8% of the patients had some risk for falls(12).

CKD favors the development of hematological disorders such as anemia, malnutrition, cardiovascular disorders and sleep disorders, and hemodialysis predisposes to electrolyte imbalance and changes in blood pressure. These factors justify high fall rates in this population(23).

It is possible that physical, sensory, and cognitive changes related to aging explain the greater risk of falls among the elderly, in relation to younger people. A study conducted in the United States reveals that elderly people with CKD have an 81% higher risk of falls when compared to younger people(24).

Another variable was the low level of education, which was also associated with a greater number of patients with the diagnosis of risk for falls. This association may be due to the difficulty in understanding the health-disease process and the prescribed treatment, and consequent non-adherence to the treatment by these patients; triggering the changes already mentioned. Health education is therefore essential during nursing care, considering the cognitive limits of patients in this group.

The diagnosis of excess fluid volume was associated with the variable treatment time and was more presented in patients with longer treatment time. Over time the patient decreases the Residual Renal Function (RRF) and therefore a greater restriction of fluids is necessary. A study carried out in the North East of Brazil, showed that patients with decreased RRF had an average of two years of haemodialysis, and those who did not have it were on treatment for an average of 7.2 years. Also, in this study, whereby patients with and without RRF were compared, it was found that patients without RRF had a greater intradialytic weight gain(25).

The decrease in RRF for patients may cause a decrease in observing water restriction, as the control of water intake will be more controlled, thus increasing the difficulty for these patients to follow the recommendations(26). A higher fluid intake than prescribed may increase cardiac overload, cause edema, electrolyte changes, pulmonary congestion, and complications in dialysis sessions, such as hypotension and cramps, due to the large withdrawal of fluids in a short period of time(21),(26)-(27).

Thus the nurse’s recognition and survey of these diagnoses, (based on defining characteristics and related and/or risk factors, and considering the individuality of each patient), is absolutely essential to direct care, not only from the technical perspective, but also taking into consideration the human responses experienced by the patient in relation to their health status.

This study has some limitations. First, the study was performed only with patients undergoing hemodialysis. Patients in other modalities of renal replacement therapy were not considered. Second, because of the cross-sectional design of our study, a causal relationship between nursing diagnoses and its association with sociodemographic and clinical factors components cannot be determined. Third, the study did not perform a multivariate analysis.

## Conclusions

The findings of this study showed the main nursing diagnoses and its association with clinical and sociodemographic variables in chronic renal patients in a Brazilian context. Future research may lead to implement care plans for the most prevalent nursing diagnoses in this type of population.

Finally, these results can provide better support to nurses in the care of patients in the treatment of hemodialysis, since they highlight important elements of the assessment. Thus, these results consider possible nursing interventions that may contribute to improvements in the clinical condition of patients, in addition to contributing to greater diagnostic accuracy.
